# Exploiting Radiation Therapy to Restore Immune Reactivity of Glioblastoma

**DOI:** 10.3389/fonc.2021.671044

**Published:** 2021-05-20

**Authors:** Mara De Martino, Oscar Padilla, Camille Daviaud, Cheng-Chia Wu, Robyn D. Gartrell, Claire Vanpouille-Box

**Affiliations:** ^1^ Department of Radiation Oncology, Weill Cornell Medicine, New York, NY, United States; ^2^ Department of Radiation Oncology, Columbia University Irving Medical Center, New York, NY, United States; ^3^ Herbert Irving Comprehensive Cancer Center, New York, NY, United States; ^4^ Department of Pediatrics, Pediatric Hematology/Oncology/SCT, Columbia University Irving Medical Center, New York, NY, United States; ^5^ Sandra and Edward Meyer Cancer Center, New York, NY, United States

**Keywords:** glioblastoma, radiotherapy, immunotherapy, antigenicity, adjuvanticity, immunosuppression

## Abstract

Glioblastoma (GBM) is among the most aggressive of brain tumors and confers a dismal prognosis despite advances in surgical technique, radiation delivery methods, chemotherapy, and tumor-treating fields. While immunotherapy (IT) has improved the care of several adult cancers with previously dismal prognoses, monotherapy with IT in GBM has shown minimal response in first recurrence. Recent discoveries in lymphatics and evaluation of blood brain barrier offer insight to improve the use of ITs and determine the best combinations of therapies, including radiation. We highlight important features of the tumor immune microenvironment in GBM and potential for combining radiation and immunotherapy to improve prognosis in this devastating disease.

## Introduction

Glioblastoma (GBM), a high-grade glial tumor, is the most frequent malignant primary brain tumor in adults (1). GBM prognosis remains dismal with a low 5-year survival rate of only 5.6% (1) and a median overall survival (OS) of approximately 18 months (2).

Immunotherapies (ITs) have long been overlooked for the treatment of central nervous system (CNS) malignancies presumably due to the long-held view of the brain as an immune-privileged compartment. However, the discovery of a dural lymphatic system (3, 4), the ability of some CNS-tissue resident cells to present antigen (5–9) and the functional characterization of the dural sinuses as an immune interface of the CNS (10) have introduced a paradigm shift whereby the brain possesses an immune-distinct tumor microenvironment (TME) that is still accessible for ITs (11–13). Since then, efforts have spurred in clinic to evaluate the efficacy of ITs in GBM (13), but the paucity of pre-existing T cells at diagnosis prevented the reactivation of anti-tumor immune responses (14–16). Notably, monotherapy with ITs have shown poor response rate in first GBM recurrence (17). In evaluation of responders to anti-PD1 monotherapy at first recurrence, patients are more likely to be Phosphatase and TENsin homolog (PTEN) wild type and have increased immune infiltration post anti-PD1 monotherapy compared to non-responders who are PTEN mutant and have low immune infiltrate both before and after IT (18). Consequently, it is critical to develop IT-based combinatorial approaches that both recruit and activate tumor-infiltrating lymphocytes (TILs).

Radiation therapy (RT) increases antigenicity and adjuvanticity of malignant cells (19), thus suggesting that RT could be used to coax T cells into GBM. Supporting this notion, several groups have reported synergism between RT and IT in preclinical models of GBM which have motivated the assessment of RT-based combinatorial approaches in Clinic (**Table 1**).

**Table 1:** Combination of immunotherapy with radiation therapy in clinical development for glioblastoma.

Target	Agent	New or Recurrent	Phase	Clinical Trial ID	Radiation regimen	Status	Notes
**PD-1**	Nivolumab	Newly diagnosed	III	NCT02617589	Standard fractionation	Active, not recruiting	Unmethylated MGMT; comparison anti-PD-1 versus TMZ each in combination with RT
**PD-1**	Nivolumab	Newly diagnosed	III	NCT02667587	Standard fractionation	Active, not recruiting	Methylated MGMT; TMZ plus RT combined with anti-PD-1
**PD-1**	Nivolumab	Newly diagnosed	I	NCT03576612	Standard fractionation	Recruiting	Neoadjuvant onclolytic adenovirus (GMCI) + TMZ
**PD-1**	Nivolumab	Recurrent	II	NCT03743662	Hypofractionated	Recruiting	Re-irradiation (6Gy x 5) +/- anti-PD-1 +/- Bevacizumab
**PD-1**	Nivolumab	Newly diagnosed	II	NCT04195139	Standard fractionation	Recruiting	Elderly patients; comparison RT+anti-PD-1 + TMZ versus standard treatment (RT+TMZ)
**PD-1**	Pembrolizumab	Newly diagnosed	II	NCT03018288	Standard fractionation	Recruiting	TMZ +/- heat shock protein (HSPPC-96)
**PD-1**	Pembrolizumab	Newly diagnosed	II	NCT03197506	Standard fractionation	Recruiting	Standard therapy (RT+TMZ) +/- anti-PD-1
**PD-1**	Pembrolizumab	Recurrent	I	NCT02313272	Standard fractionation	Active, not recruiting	Bevacizumab and RT (6Gy x 5) +/- anti-PD-1
**PD-1**	Pembrolizumab	Newly diagnosed	II	NCT03899857	Standard fractionation	Recruiting	standard treatment (RT+TMZ) + anti-PD-1
**PD-1**	Pembrolizumab	Newly diagnosed	I	NCT02287428	Standard fractionation	Recruiting	Unmethylated MGMT; RT+anti-PD-1+NeoAntigen Vaccine
**PD-1**	Pembrolizumab	Newly diagnosed	I	NCT03426891	Standard fractionation	Recruiting	Standard therapy (RT+TMZ) +/- HDAC inhibitor (Vorinostat) +/- anti-PD-1
**PD-1**	Pembrolizumab	Recurrent	II	NCT03661723	Hypofractionated	Recruiting	Re-irradiation (7Gy x 5) per week for 2 weeks +/- Bevacizumab
**PD-1 and CTLA-4**	Nivolumab and Ipilimumab	Newly diagnosed	II	NCT03367715	Hypofractionated	Recruiting	Unmethylated MGMT; RT (6Gy x 5) + anti-PD-1 + anti-CTLA4
**PD-1 and CTLA-4**	Nivolumab and Ipilimumab	Newly diagnosed	II/III	NCT04396860	Standard fractionation	Recruiting	Unmethylated MGMT; comparison standard treatment (RT+TMZ) versus RT+anti-PD-1+anti-CTLA-4
**PD-1 and IDO**	Nivolumab and BMS-986205	Newly diagnosed	I	NCT04047706	Standard fractionation	Recruiting	Standard treatment (RT+TMZ) +/- anti-PD-1 +/- IDO inhibitor
**PD-L1**	Durvalumab	Newly diagnosed and recurrent	II	NCT02336165	Standard fractionation	Active, not recruiting	Bevacizumab
**PD-L1**	Durvalumab	Recurrent	I/II	NCT02866747	Hypofractionated	Recruiting	RT (8Gy x 3)
**PD-L1**	Atezolizumab	Newly diagnosed	I/II	NCT03174197	Standard fractionation	Active, not recruiting	Standard treatment (RT+TMZ) +/− anti-PD-L1
**PD-L1**	Avelumab	Newly diagnosed	II	NCT02968940	Hypofractionated	Completed	IDH mutant; RT (6Gy x 5)
**PD-L1**	Avelumab	Newly diagnosed	II	NCT03047473	Standard fractionation	Active, not recruiting	Standard treatment (RT+TMZ) +/− anti-PD-L1
**PD-L1**	Avelumab	Recurrent	II	NCT03291314	Standard fractionation	Completed	Standard treatment (RT+TMZ) + anti-PD-L1 + tyrosine kinase inhibitor (axitinib)
**GM-CSF**	Sargranostim	Newly diagnosed	II	NCT02663440	Hypofractionated	Unknown	RT (regimen not specified) + TMZ + GM-CSF
**GM-CSF and poly I:C**	Sargranostim and Hiltonol	Recurrent	I	NCT03392545	Not specified	Recruiting	RT + GM-CSF and poly I:C
**GM-CSF and tetanus-diphtheria toxoid (Td)**	GM-CSF and Td	Newly diagnosed	II	NCT03927222	Standard fractionation	Recruiting	Unmethylated MGMT; Standard treatment (RT+TMZ) + Td + GM-CSF
**TGF-β**	Galunisertib	Newly diagnosed	I/II	NCT01220271	Standard fractionation	Completed	Standard treatment (RT+TMZ) +/− anti-TGF-β
**IDO**	Indoximod	Newly diagnosed	I/II	NCT02052648	Hypofractionated	Completed	TMZ +/− bevacizumab +/− IDO inhibitor +/− RT (5.5 × 5 Gy)
**CXCR4**	Plexirafor	Newly diagnosed	I/II	NCT01977677	Standard fractionation	Completed	Standard treatment (RT+TMZ) +/− CXCR4 inhibitor
**CSF1R**	Pexidartinib	Newly diagnosed	I/II	NCT01790503	Standard fractionation	Completed	Standard treatment (RT+TMZ) +/− CSF1R inhibitor
**IGF-1R**	IGV-001	Newly diagnosed	Iib	NCT04485949	Standard fractionation	Not yet recruiting	Standard treatment (RT+TMZ) +/− IGV-001 cell immunotherapy
**PD-L1**	Atezolizumab	Recurrent	II	NCT04729959	Hypofractionated	Not yet recruiting	IDH1 wild type; PD-L1 inhibitor; tocilizumab; RT

Here will we discuss the unique immune system of the central nervous system (CNS), the immunosuppressive TME of GBM and how RT can restore the sensitivity of GBM to modern IT by modulating systemic and local anti-tumor immunity.

## The Unique Immune System of the Central Nervous System

The traditional dogma of the brain as an immune-privileged organ was initiated by pioneer work from Murphy in the 1920s, demonstrating successful growth of mouse sarcoma after their implantation into the brain while rejection of these tumors was observed when transplanted in the periphery (20). Later on, these findings were confirmed with seminal work from Medawar, in the 1940s, which similarly demonstrate a high propensity of tumor engraftment in the brain parenchyma as opposed to tumor transplant in peripheral organs (21). Of notice, when first transplanted in peripheral organs before their implantation into the brain, these tumors were successfully rejected, thus suggesting that the activation of the immune system in the periphery can generate tumor rejection into the brain (21). Consequently, the fact that brains were unable to elicit anti-tumor immune responses by itself led to the concept of the immune privilege of the CNS.

Since then, studies have revealed that the immune privilege status of the CNS is overstated. Notably, the description of the afferent mechanism for CNS engagement in regional lymphatic (22–24) together with the discovery of the glymphatic (glial-lymphatic) system that links the parenchyma and the interstitium to the cerebrospinal fluid (CSF) spaces, started to challenge the concept of the brain as immunologically silenced.

Another breakthrough in the field of brain immunology was the identification of a functional meningeal lymphatic network that enables the drainage of immune cells, macromolecules and fluids from the CNS to the deep cervical lymph nodes (dcLN) (4, 25). This dural lymphatic system provides a physical connection for CSF-derived antigens to gain access to dcLN for priming and activation of T cells. Consequently, meningeal lymphatic vessels are critical regulators of drainage and immune surveillance, a notion that has been demonstrated in the context of GBM (26, 27). More recently, the dural sinuses were identified as a neuro-immune hub where circulating T cells can assess the brain and CSF-derived antigens to enable immune surveillance (10).

Given the complex lymphatic circuitry and the unique sites of neuro-interface of the CNS, the brain can no longer be perceived as an immune-privileged organ, but rather as an immune-distinct and highly immunosuppressive environment.

This concept is reinforced by the ongoing challenge of the efferent arm of CNS immunity. Indeed, the blood brain barrier (BBB), a structure composed of capillary tight junctions and astrocyte cell projections (aka astrocytic feet or “glia limitans”) (28, 29), is thought to serve as a filter of the transit of molecules and immune cells between the brain and the systemic circulation. Some strategies to overcome the BBB have been explored, including the usage of nanoparticles, convection enhanced delivery, and non-invasive focused ultrasound and have achieved promising results in preclinical models (30–34). However, the recent demonstration of T cells infiltration and immune surveillance of the brain challenge the long-held view of BBB as an hermetic barrier to immune cell trafficking and suggest that the CNS is accessible to immune cells (35–37).

Aside distinct afferent and efferent circuits of CNS immunity, tissue-resident myeloid cells are another unique feature of brain immunity (38). This population is mainly composed of microglia (or tissue-resident macrophages) that originate from the yolk sac and migrate into the brain during embryonic development (39). The function of microglia is to assess the brain parenchyma and to maintain immunological homeostasis by responding to signals consistent with tissue damage, inflammation, or the presence of pathogens (40, 41). Such activation of the microglia leads to an increase capacity of antigen presenting functions as well as its phagocytic properties, suggesting that microglia serves as the resident antigen-presenting cells of the CNS (5, 9).

**Figure 1:**
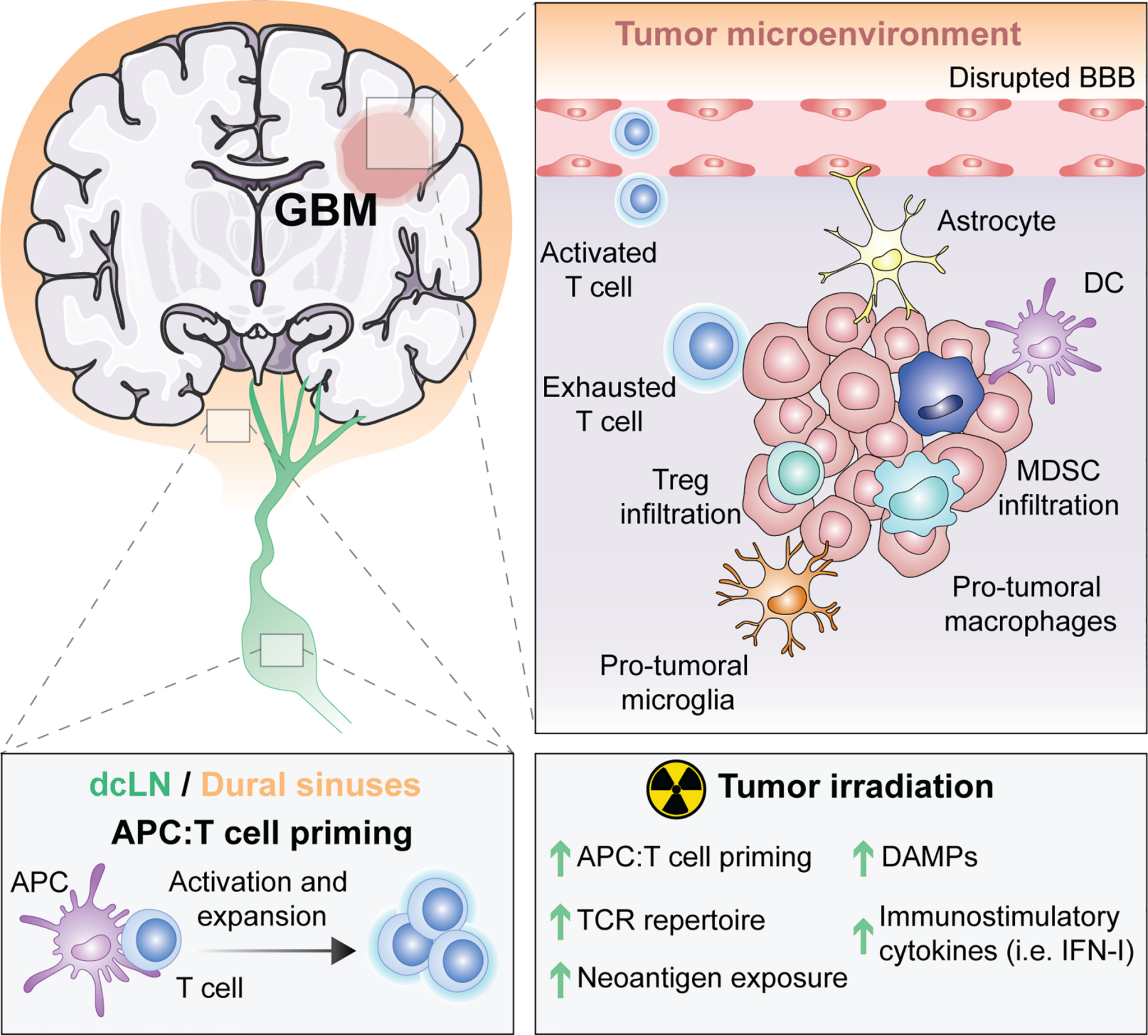
The unique immune response in GBM and its modulation by RT. For many years, the central nervous system (CNS) was thought to be excluded from immune surveillance. However, it is now known that the CNS is not isolated from activated T cells and that CNS antigens can be presented locally or peripherally in the draining cervical lymph nodes or the dural sinuses. Diverse types of antigen presenting cells (APCs) exist within glioblastoma (GBM), including microglia, macrophages, astrocytes and classic APCs such as dendritic cells (DCs). APCs that have captured tumor antigens can present to naïve T cells, leading to their activation and expansion. Activated T cells migrate into the brain through a disrupted blood brain barrier (BBB), but once in the tumor microenvironment (TME) they differentiate into exhausted T cells. Within the TME, there are immunosuppressive regulatory T cells (Tregs), myeloid derived suppressor cells (MDSC), reactive astrocytes and pro-tumoral macrophages and microglia. Radiotherapy (RT), the standard of care for GBM, induces the exposure of tumor neoantigens and increases the T cell receptor (TCR) repertoire. Moreover, tumor irradiation promotes the release of danger associated molecular patterns (DAMPs) and type I interferon (IFN-I), which stimulate APCs cross-priming of T cells. All of these suggest that RT can be used to overcome GBM immunosuppression to optimally prime anti-tumor immunity.

Thus, the unique features of the brain from its drainage to its tissue resident microglial cells (**Figure 1**) suggest that immune responses in the CNS are possible. However, the immune singularity of the brain calls for a better understanding of CNS immunity to optimally generate anti-tumor immunity against brain malignancies.

## The Immune Suppressive Microenvironment of Glioblastoma

A major obstacle to anti-tumor immune responses against GBM is its highly immunosuppressive TME (**Figure 1**).

Among key contributors to GBM immunosuppression, tumor-associated macrophages cells (TAMs) account for 30% to 50% of the tumor mass (42, 43). TAMs are usually pro-tumorigenic, and their accumulation correlate with tumor grade and poor prognosis (44–46). The recruitment and function of TAMs is modulated by GBM-secreted factors, such as the chemo-attractants stromal cell-derived factor 1 (SDF1) (47, 48), C–C motif chemokine ligand 2 (CCL2) (49, 50) and the colony-stimulation factor 1 (CSF1) (51). TAMs promote immunosuppression by the production of arginase, transforming growth factor-beta (TGFβ), interleukin (IL)-10 and IL-6, among others which collectively inhibit both the innate and adaptive immune systems with suppression of NK activity and T cell activation and proliferation (52–55).

Another mechanism responsible for immunosuppression and ultimately the lack of response of IT strategies in GBM patient is the low representation of T cells in the tumor. Studies have demonstrated that T cells influx in GBM is offset as a result of (1) reduced T cells production subsequent to thymic involution (56), (2) increased expression of the programmed death ligand-1 (PD-L1) (57), (3) loss of surface spingosine-1-phosphate receptor 1 (S1P1) in brain tumors to sequester T cells in the bone marrow (58) and (4) CD68+ microglia lose MHC-II (i.e. heterogeneity human leukocyte antigen (HLA)-DR isotype; HLA-DR) expression in a PTEN dependent fashion (18).

Despite these major obstacles, some T cells can successfully infiltrate intracranial tumors and have been shown post IT in patients who respond (18). However, infiltrating T cells are more likely to be dysfunctional and express markers of exhaustion like programmed cell death (PD-1), lymphocyte-activation gene 3 (LAG3) and T-cell immunoglobulin and mucin-domain containing-3 (TIM-3) (59–61). Importantly, a large proportion of T cells infiltrating GBM are regulatory T cells (Tregs) that co-expressed checkpoint inhibitors including cytotoxic T-lymphocyte-associated protein 4 (CTLA-4) and PD-1 (62). Treg is a subset of CD4 T cells that express the transcription factor forkhead box protein3 (Foxp3) (63, 64). These cells suppress CD8 T cells activation by the secretion of immunosuppressive cytokines, namely TGFβ and IL-10 (65, 66). GBM attract Tregs from the periphery to the local TME by soluble factors, such as GBM-derived CCL22, CCL2, and TGFβ, to promote immunosuppression (66–69).

Overall, these findings underscore that not only do intracranial tumors display high infiltration of immunosuppressive cells but they also secrete factors that limit T cell responses against GBM.

The Cancer Genome Atlas (TCGA) has identified four subtypes of GBM (i.e. proneural, neural, classical, and mesenchymal), based on mutations that drive proliferation and survival of GBM (70). Consequently, the genetic heterogeneity of GBM predicts for a great mutational load, one of the favorable biomarkers for successful IT. However, GBM are characterized by a relatively low mutational burden (71, 72), suggesting that GBM display limited somatic mutations for the T cells to target and ultimately lead to a restricted efficacy of IT when used as monotherapy.

Defects in the antigen presentation machinery, such as downregulation or loss of HLA class I, have also been reported in GBM patients (73). More specifically, microglia antigen-presenting cells (9, 74) present a downregulation of the major histocompatibility class I (MHC-I) due to immunosuppressive cytokines (e.g. TGFβ and IL-10) that emanate from the TME (75).

Therefore, low presence of antigens combined with defective presentation, represent an additional challenge to mount effective T cell responses against GBM.

Metabolic alterations of GBM is an emerging immune resistance mechanism (76). Notably, a recent study comparing the metabolic reprogramming of GBM patient samples with low-grade astrocytoma identified that variations in tryptophan, arginine, prostaglandin, and adenosine pathways might be responsible for the accumulation of Tregs and pro-tumorigenic TAMs in GBM (77). Moreover, activation of the mammalian target of rapamycin (mTOR) pathway in microglia promoted tumor growth and immune evasion in murine GBM (78). Therefore, targeting metabolic liabilities of intracranial tumors represents a promising strategy to overcome immunosuppression.

## Radiotherapy to Restore the Sensitivity of Glioblastoma to Immunotherapy

The complexity of brain immunity combined with the immunosuppression exerted by the TME in brain tumors call for innovative approaches to break immune tolerance of brain malignancy.

One appealing strategy is to exploit the immuno-stimulatory properties of RT to generate an *in situ* tumor vaccine and the subsequent recruitment of effector T cells into GBM; a vital component for the efficacy of modern IT (**Figure 1**).

RT has been acknowledged as a potent immune adjuvant over the past two decades with major preclinical data demonstrating that RT promotes tumor specific T cell responses (79, 80). However, the concept of RT as an immune response modifier (IRM) was initiated forty years ago by Stone who demonstrated that responses to RT were impaired in the absence of T cells (81). While these findings were ignored for a long time, the breakthrough of ITs restimulated interest in exploiting the immunogenic properties of RT to expand the fraction of cancer patients that can benefit from IT. Since then, studies from experimental models have provided mechanistic insight pertaining to the ability of RT to stimulate the immune system. Notably, two main processes were found essential (but not mutually exclusive) to convey immunogenicity of an irradiated tumor: (1) the engagement of an immunogenic cell death (ICD) (82–85) and (2) the induction of type I interferon (IFN-I) (86–88). ICD is identified by the spatial and temporal occurrence of three damage-associated molecular pattern (DAMPs) molecules, namely the pre-apoptotic exposure of calreticulin (CRT) on the cell surface (89), the active secretion of ATP (82, 83, 89–92) and the release of the non-histone nuclear protein High Mobolity Group Box 1 (HMGB1) (82).

Activation of IFN-I response is essential for T cell priming and is a consequence of the recognition of cytosolic double stranded (ds) DNA by the nucleic acid sensor (NAS) CGAS (i.e. cyclic GMP-AMP synthase) to engage stimulator of the interferon genes (STING) pathway in irradiated cells as well as in dendritic cells (DC) (87, 88, 93–98). The source of cytosolic DNA is currently being debated with reports indicating that micronuclei formed by mitotic defects (99–101) and/or the autophagy-dependent release of mitochondrial dsDNA (86).

Nevertheless, RT-induced IFN-I response is not restricted to cytoplasmic dsDNA sensing. Notably, recent studies have demonstrated that cytoplasmic recognition of dsRNA by the retinoic acid inducible gene I (RIG-I)-like receptors (RLRs) led to IFN-I post RT (102, 103). Cytosolic dsRNA sensing involves three RLR sensors, namely RIG-I, melanoma differentiation-associated gene 5 (MDA5), and laboratory of physiology and genetics 2 (LGP2 or DExH-box helicase 58; DHX58) (104, 105). A recent preclinical study, reported that host LGP2 was essential for optimal anti-tumor control of irradiated murine colorectal tumors (103). Consequently, the activation of RT-induced IFN-I is the result of DNA recognition by the CGAS-STING pathway but is also subsequent to RNA sensing by the RLR family. Whether these mechanisms are initiated in irradiated GBM remains unknown, but current data suggests that activation CGAS-STING in myeloid cells is important for anti-tumor immunity against this tumor type (106, 107).

Other major immunogenic features of RT is to shape the T cell receptor (TCR) repertoire of TILs (108–112) and to expose immunogenic mutations to the immune system (113). A detailed discussion describing the mechanisms responsible for the increase of antigenicity in irradiated tumors can be found elsewhere (114).

While the capacity of RT to generate similar mechanisms in the brain remains to be investigated, evidence of MHC-I upregulation and increase of antigen presentation from brain irradiation was described (115). More importantly, it was reported that personalized neoantigen vaccine generates intratumoral T cell responses in GBM patients, suggesting that RT-induced immunogenic mutation exposure is a promising strategy to treat intracranial tumors (116).

The impact of the isocitrate dehydrogenase 1 (IDH1) mutation together with the methylation status of O6-methylguanine-DNA methyltransferase (MGMT) on RT-induced anti-tumor immunity against GBM is unclear. However, the fact that neoantigen derived from mutant IDH1 can promote anti-tumor CD4+ T-cells and antibody responses in glioma together with the ability of RT to expose neoantigens, suggest that IDH1 mutated GBM patients might better respond to the RT-IT combinations as opposed to patients with wild-type IDH1 tumors (117).

Altogether, mechanistic insights pertaining to the immunogenic role and function of ionizing radiation elevated the use of RT as a partner to IT in multiple cancer including GBM. Some RT-IT combination are already assessed in preclinical models of GBM as well as in clinic (**Table 1**). For instance, focal irradiation improved the survival of GBM-tumor bearing mice treated with anti-PD-1 (118, 119), anti-CTLA-4 + 4-1BB activation (120), dual TIM-3 and PD-1 blockade (121) and anti-GITR (glucocorticoid-induced TNFR family related gene) (122). Underscoring the potential of RT to promote GBM-targeted T cells responses, all of these studies reported an increase in T cell infiltration and some even documented long-lasting immune memory responses against GBM.

Importantly myeloid cells expressing the colony-stimulating factor-1 receptor (CSF-1R) (or TAM-CSF-1R+ cells) were recently found altered during the time-course of anti-GBM therapy. Notably, RT was described to promote recurrence-specific phenotypes in microglia and monocyte-derived macrophages (123). GBM tumor bearing mice treated with the combination of anti-CSF-1R with focal RT experienced increase in survival, thus indicating that CSF-1R targeting is a promising strategy for irradiated GBM (123).

Along similar lines, targeting PD-L1 expressing tumor associated myeloid cells in combination with dinaciclib, a cyclin-dependent kinase inhibitor, extended survival of mice bearing irradiated GBM tumors (124).

## Clinical Translation and Challenges

A widespread interest of RT-based immuno-oncology combinations has spurred in Clinic due to the mounting evidence highlighting the role RT as an immune adjuvant. However, the clinic translation of experimental models turn out to be more challenging than anticipated due to several of host-responses to RT. Notably, mounting evidence highlight the critical aspect of the choice of radiation fractionation and regimen to elicit anti-tumor immunity. Consequently, the impact of RT planning and delivery must be considered including: absolute dose, dose-per-fraction, low dose spread, path of radiation delivery, and the effects of radiation cell kill. Radiation dose fractionation and dose per fraction has shown to differentially affect immune cells and the TME. For instance, radiation dose-dependent responses can be elicited on T-effector cells versus Tregs, macrophages, and TME regulation through TREX1-STING-IFN signaling (87, 125–128). The optimal radiation dose and regimen together with the best sequencing between IT and RT remains elusive (19, 129, 130).

Nevertheless, ongoing clinical trials assessing the combination of IT with either standard fractionation or hypofractionation regimen in CNS diseases (**Table 1**) may provide some indication on the optimal radiation regimen and sequencing of IT to generate GBM-targeted anti-tumor immune responses.

Another major limitation to RT-induced anti-tumor immunity is the activation of latent TGFβ that stem for the TME. TGFβ activation by RT promotes immunosuppression (131) and therefore represents a major challenge for the translation of RT-IT combinations. Nevertheless, cooperative effects of TGFβ blockade with focal RT has shown some promises in patients with metastatic breast cancer (132, 133), which underscore that blocking TGFβ in the context of RT might be required to elicit potent anti-tumor immunity.

There are many emerging ionizing radiation technologies that may further add to the immune modulatory effects including ultras-fast dose-rate radiotherapy (FLASH-RT) and particle therapy (proton and carbon ion therapy) (134). While preclinical studies hold great promises to generates anti-tumor immunity against FLASH-irradiated GBM (135), additional investigations are required to define the immunogenic properties of FLASH radiation, especially in the context of brain malignancies.

Overall, to achieve clinical translation for patient care, increase knowledge of the interplay between radiation responses of the host and immunosuppression must be investigated.

## Conclusion

Although to date, the clinical trials assessing the efficacy of IT have been disappointing, the results from preclinical studies are very encouraging for the success of RT-IT combinations in treating GBM. Different strategies adapted from experimental models are currently being investigated to harness the immense potential of combining RT with IT (**Table 1**). As a scientific community, we strongly await the data from these ongoing clinical trials. Further efforts to understand the effect of RT in TME of GBM may uncover novel avenues to optimally combine RT with IT to generate an *in situ* vaccination against GBM. However, given the complexity of the brain immunity, together with the immunosuppression of GBM, it is likely that multiple targets will be required to eliminate irradiated GBM.

## Author Contributions

All authors (MD, OP, CD, C-CW, RG, and CV-B) contributed to article writing and editing. All authors contributed to the article and approved the submitted version.

## Funding

OP is supported by National Cancer Institute (NCI) Stimulating Access to Research in Residency (StARR) Award, supplement to the Columbia Cancer Research Program for Resident Investigators (CAPRI, R38CA231577). C-CW is supported by the Gary and Yael Fegel Family Foundation, the Star and Storm Foundation, the Matheson Foundation (UR010590), and a Herbert Irving Cancer Center Cancer Center Support Grant (P30CA013696). RG is supported by Swim Across America and Hyundai Hope on Wheels Hope Scholar Award. CV-B is supported by a startup grant from the Department of Radiation Oncology at Weill Cornell Medicine and a Brain Cancer Research Investigator Grant from B*CURED.

## Conflict of Interest

The authors declare that the research was conducted in the absence of any commercial or financial relationships that could be construed as a potential conflict of interest.
